# Network analysis of the symptoms of posttraumatic stress disorder in patients undergoing chemotherapy for colorectal cancer

**DOI:** 10.3389/fpsyt.2024.1505518

**Published:** 2025-01-08

**Authors:** Yi Zhou, Wen Teng, Liang Luo, Ping Zhou, Guidi Fan, Haiying Liu

**Affiliations:** ^1^ Wuxi School of Medicine, Jiangnan University, Wuxi, China; ^2^ Department of Intensive Care Unit, Wuxi No.2 People’s Hospital (Jiangnan University Medical Center), Wuxi, China; ^3^ Department of General Surgery, Wuxi No.2 People’s Hospital (Jiangnan University Medical Center), Wuxi, China; ^4^ Department of Infection Management, Wuxi No.2 People’s Hospital (Jiangnan University Medical Center), Wuxi, China

**Keywords:** colorectal cancer, chemotherapy, PTSD, network analysis, symptom structure

## Abstract

**Objective:**

In this study, we examine the network structure of posttraumatic stress disorder (PTSD), including core symptoms and strong edges in patients undergoing chemotherapy for colorectal cancer in China, and lay the groundwork for targeted psychological interventions for these patients.

**Methods:**

This study included 360 colorectal cancer patients receiving chemotherapy at a third-class hospital in Wuxi, China, from November 2023 to June 2024. The severity of each item of PTSD was assessed using the DSM-5 Checklist (PCL-5). A network analysis approach was conducted in R to pinpoint core symptoms and investigate notable edge connections within the network.

**Results:**

The accuracy and stability of the PTSD network structure model were relatively good, and the results indicated that robust connections emerged between avoidance of thoughts and avoidance of reminders, hypervigilance and exaggerated startle response, and loss of interest and detachment. The most central node was emotional cue reactivity, which was more closely connected with other symptoms, while self-destructive/reckless behavior was the lowest central node.

**Conclusion:**

Emotional cue reactivity was proved to be the most prominent symptom in the PTSD symptom network in colorectal cancer patients treated with chemotherapy, and targeting it in interventions could lead to substantial improvements.

## 1 Introduction

Colorectal cancer (CRC), recognized as a malignant tumor, is one of the most common cancers in the world. In 2022, the global incidence of new CRC cases accounted for 10.7% of all cancer types, making it the second most common cause of cancer-related mortality, following lung cancer (1). Moreover, CRC incidence and mortality rates in China continue to rise, surpassing the global average (2). After experiencing a series of significant stressors, including a cancer diagnosis and chemotherapy, CRC patients often encounter negative emotions (3), such as depression and anxiety. They may even develop posttraumatic stress disorder (PTSD) (4). Research has reported that the positive rate of PTSD in CRC patients ranges from 11.2% to 32.3% (5, 6). PTSD mainly manifests in intrusive traumatic experiences, avoidance, negative cognitive and emotional changes, and persistent hypervigilance (4). Under prolonged psychological stress from traumatic events, PTSD patients may experience infections (7), pain (8), somatic symptoms (9), and changes in systemic immune function, which seriously affect their quality of life and prognosis (10).

PTSD clinical symptoms are diverse, with their interactions potentially influencing the progression of the disorder (11). Previous research often treated PTSD as a whole (12, 13), assuming that all symptoms or items of PTSD are equally important. Researchers typically use the total symptom score to assess the severity and as an indicator of treatment effectiveness, ignoring the complex links and interactions among the symptoms.

Network analysis is a research approach to investigating and comprehending the symptoms (nodes)within a network and the relationships (edges)that connect them. It shifts the focus from the disease to the uniqueness of each symptom and its internal connections (14). Through network analysis, we plan to identify the most influential central symptoms and strongly associated edges, expecting that this will help to reveal the underlying structure of disease symptoms and enhance our understanding of how symptoms interact (15).

To date, network analysis has become more prevalent in examining PTSD symptoms (16–18). Individuals who have experienced similar traumatic events display specific shared response patterns, which may create a tightly linked PTSD network, increasing their vulnerability to developing PTSD (17). It is important to note that various types of trauma and demographic groups can result in differences in the features of the network framework (16, 19–21). For example, hypervigilance frequently appears as the primary symptom among adults who have suffered from sudden natural disasters like earthquakes (21). In veterans exposed to war environments, the core PTSD symptoms are flashbacks and emotional cue reactivity (19).

Currently, the network structure and core symptoms of PTSD in Chinese colorectal cancer patients in chemotherapy treatment remain unclear. Given that the network characteristics of PTSD vary by type of trauma and population, this suggests that findings from previous studies may not apply to the colorectal cancer group. Therefore, we will carry out network research on PTSD in colorectal cancer patients to explore their unique response patterns.

## 2 Methods

### 2.1 Participants

This research utilized a convenience sampling method involving CRC individuals who fulfilled the following criteria. The study occurred in the general surgery and oncology wards of a tertiary medical facility in Wuxi, Jiangsu Province, China, from November 2023 to June 2024. According to the sample size estimation of network analysis, In a twenty-node network, 210 parameters (twenty threshold parameters and 20×19/2 = 190 pairwise association parameters) need to be estimated (22). Therefore, the minimum sample size required for this study is 210 cases. This research received approval from the Ethics Committee at Wuxi No.2 People’s Hospital (Ethics number: 2023Y-172). Participation was voluntary, and consent was obtained from all participants. The criteria for inclusion were: 1) a diagnosis of colorectal cancer; 2) aged 18 years or older and sufficiently clear-minded to possess basic reading comprehension skills; 3) currently undergoing chemotherapy; 4) aware of their diagnosis and condition and willing to participate in the research. The criteria for exclusion were: 1) cancer diagnosis not exceeding one month; 2) history of mental illness; 3) presence of severe comorbidities that hinder cooperation (such as ardiac, pulmonary or renal insufficiency); 4) experience of other significant traumatic events in the past six months (such as natural disasters or loss of a close relative); 5) presence of other malignant tumors; 6) currently participating in other intervention studies.

During the study, the researcher explained the study’s objective to participants, gathered their consent, and distributed paper questionnaires. The researcher offered standardized guidance to assist participants with questions during the questionnaire’s completion. Once the questionnaire was filled out, the researcher promptly gathered and reviewed the questionnaires. A total of 378 questionnaires were distributed. After excluding 7 patients with a psychiatric history, 3 patients who had experienced other traumatic events within the past six months, and 8 patients with other malignant tumors, 360 valid responses were collected.

### 2.2 Measures

#### 2.2.1 Demographics and disease characteristics

A self-report questionnaire was used to collect sociodemographic and clinical information. (For example, gender, age, occupational status, marital status, educational level, place of residence, monthly household income, tumor type, cancer stage, enterostomy, surgery, radiotherapy, and length of diagnosis).

#### 2.2.2 The PTSD checklist for DSM-5(PCL-5)

Consisting of 20 items, the PTSD Checklist for DSM-5 (PCL-5) is a self-report measure that evaluates PTSD symptoms based on DSM-5 criteria (23). In this study, we used the Mandarin version of the PCL-5 (24), which has a Cronbach’s α of 0.881. This tool evaluates four PTSD symptom clusters defined by the DSM-5: intrusions, avoidance, negative alterations in cognition and mood (NACM), and hyperarousal. Participants used a 5-point Likert scale, from 0 (not at all) to 4 (significantly). They rated how much specific symptoms had affected them over the past month. Scores can vary from 0 to 80, where a score of 33 or higher indicates significant PTSD and serves as a diagnostic reference.

### 2.3 Data analysis

We conducted descriptive statistics for demographic characteristics, clinical information, and PTSD scores using SPSS 29.0. Next, we conducted symptom network estimation, centrality measurement, and accuracy and stability assessments using R packages in R version 4.4.1.

#### 2.3.1 Network estimation

Performed with the qgraph package in R was the estimation of the PTSD network. The Least Absolute Shrinkage and Selection Operator (LASSO) regression, combined with the Extended Bayesian Information Criterion (EBIC), was utilized to shrink the edges in the network (25). This approach aimed to remove relatively weak connections, enhancing the sparsity and clarity of the symptom network and making the results easier to interpret. Shorter and thicker edges in the network represent stronger associations between nodes. Blue edges signify positive correlations, while red edges denote negative correlations.

#### 2.3.2 Centrality measurements

Each node’s expected influence, strength, closeness, and betweenness were computed using the qgraph package in R. Expected influence indicates the aggregate of the edge weights that connect a node to its adjacent nodes, accounting for positive and negative associations (15). Strength refers to the total absolute values of edge weights tied to that node, demonstrating the degree of direct connections. Closeness and betweenness reflect the extent of indirect connections for the node. This study will focus on the expected influence metric, the most reliable, stable, and accurate indicator compared to other centrality measures (18). Higher EI represents the node’s increased centrality, signifying its greater importance.

#### 2.3.3 Stability and accuracy analysis

The stability and accuracy of the networks were analyzed through the bootnet package in R. First, accuracy was estimated by calculating 95% confidence intervals (CIs) using non-parametric bootstrapping (nBoots = 1000). Narrower CIs indicate more reliable edge weight estimates, while less overlap between CIs suggests greater accuracy in the network edge weights (22). Subsequently, the stability of the centrality measures was evaluated by calculating the Correlation Stability Coefficient (CS-C) through the subset bootstrap method. The CS-C reflects the highest percentage of cases that can be omitted while maintaining a correlation with 95% confidence, with a value ideally above 0.50 and not lower than 0.25 to ensure stability (26).

## 3 Results

### 3.1 Sample descriptive

**Table 1** shows the participants’ characteristics. A total of 360 colorectal cancer chemotherapy patients participated in this study, with males accounting for 55.6% (200 cases) and females for 44.4% (160 cases). The mean age of the patients was 66.39 ± 10.46 years. (see **Table 1** for details). The PCL-5 score for these patients was 21.07 ± 10.15, with 52 patients (14.4%) scoring ≥33 on the PCL-5. The scores for each scale item are shown in **Table 2**.

**Table 1:** Characteristics of the participants(n=360).

Variables		n(%) or Mean ± SD
Gender	male	200(55.6)
	female	160(44.4)
Age(years)	<45	14(3.9)
	45~59	68(18.9)
	60~74	194(53.9)
	≥75	84(23.3)
Educational level	Primary school and below	81(22.5)
	Junior high school	146(40.6)
	High school or technical secondary school	90(25)
	Junior college or above	43(11.9)
Occupational status	Be employed	52(14.4)
	Unemployed	95(26.4)
	Retired	213(59.2)
Marital status	Unmarried	2(0.6)
	Married	311(86.4)
	Divorce	6(1.7)
	Widowed	41(11.4)
Place of residence	Town	106(29.4)
	City	254(70.6)
Monthly household income (in RMB)	<3000	88(24.4)
	3000~5000	180(50)
	>5000	92(25.6)
Tumor type	Colon cancer	234(65)
	Rectal cancer	126(35)
Enterostomy	No	265(73.6)
	Temporary stoma	78(21.7)
	Permanent stoma	17(4.7)
Cancer stage	I/II	142(39.44)
	III	103(28.6)
	IV	107(29.7)
	Missing	4(1.1)
History of Surgery	Yes	309(85.83)
	No	51(14.17)
History of radiotherapy	Yes	37(10.28)
	No	323(89.72)
Length of diagnosis(month)	≤12	214(59.44)
	>12	146(40.56)

**Table 2:** Descriptions and Univariate Statistics for Network Nodes.

Node names	Symptoms	Mean	SD
B1	Intrusive thoughts	1.48	1.007
B2	Nightmares	0.62	0.9
B3	Flashbacks	0.39	0.578
B4	Emotional cue reactivity	1.46	0.972
B5	Physiological cue reactivity	0.31	0.596
C1	Avoidance of thoughts	1.79	1.016
C2	Avoidance of reminders	1.74	1.033
D1	Trauma-related amnesia	0.69	0.833
D2	Negative belief	1.19	0.874
D3	Blame of self or others	0.88	0.851
D4	Negative trauma-related emotions	1.23	0.895
D5	Loss of interest	1.61	1.143
D6	Detachment	1.27	1.121
D7	Restricted affect	1.25	0.848
E1	Irritability	1.12	1.016
E2	Self-destructive/reckless behavior	0.16	0.378
E3	Hypervigilance	0.7	0.854
E4	Exaggerated startle response	0.77	0.882
E5	Difficulty concentrating	0.6	0.717
E6	Sleep disturbance	1.8	1.191

### 3.2 The network estimation

**Figure 1:**
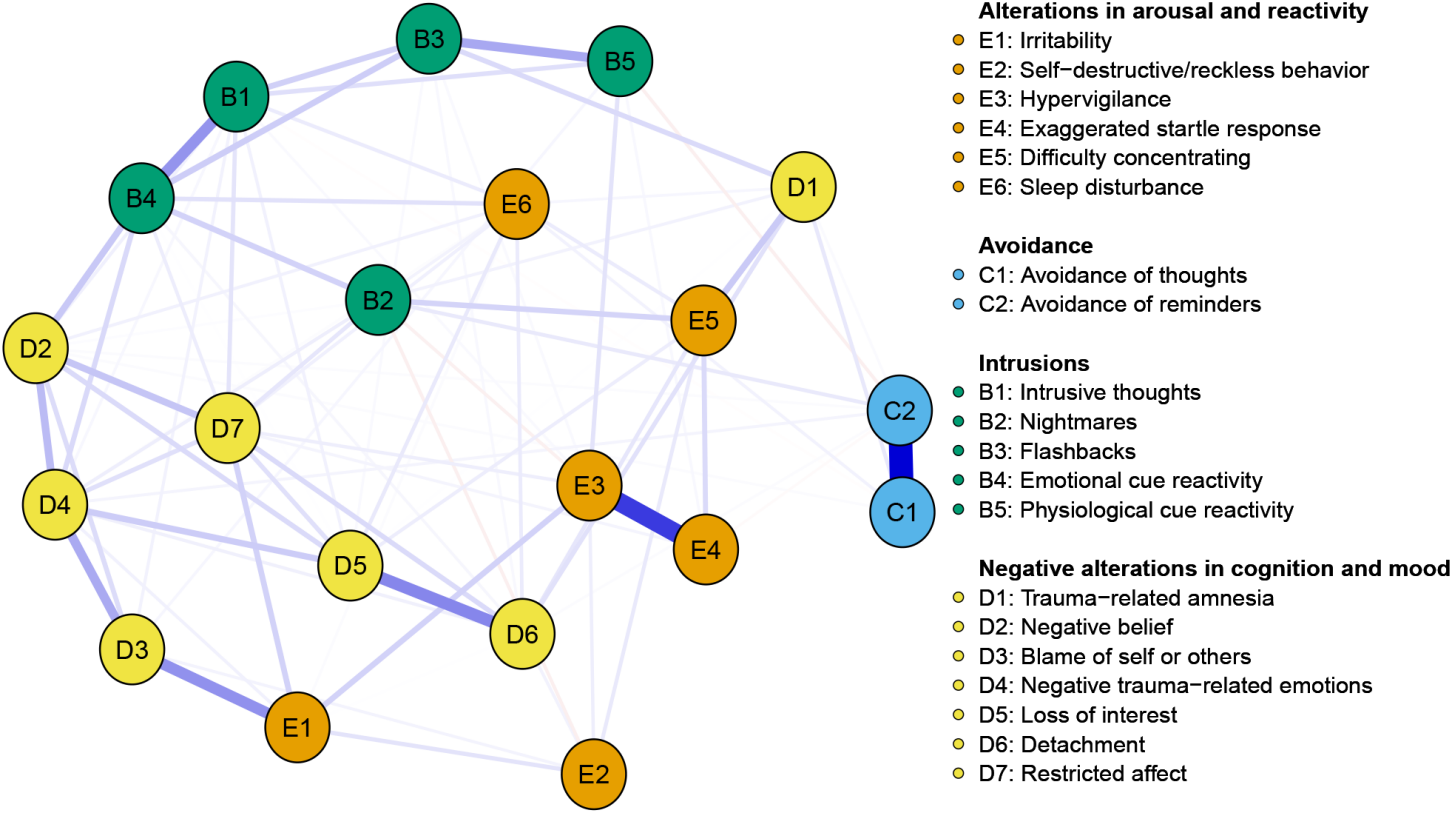
Network structure model of colorectal cancer patients receiving chemotherapy. The blue edges represent positive correlations, and the red represents negative correlations. Edge thickness represents the strength of the connection between symptoms.

**Figure 1** shows the network structure diagram of PTSD in chemotherapy patients with colorectal cancer, which includes 20 nodes and a total of 85 non-zero edges (network density is 0.45) with an average edge weight of 0.044. There are 79 positive blue edges (93%) and 6 negative red edges (7%)—A more robust correlation exists when the lines connecting two network nodes are shorter and thicker. The associations of symptoms within dimensions are slightly more potent than those between dimensions. The strongest edge weights in the network structure appear between avoidance of thoughts(C1) and avoidance of reminders(C2)(edge = 0.85), hypervigilance(E3) and exaggerated startle response(E4)(edge = 0.66) and loss of interest(D5) and detachment(D6)(edge = 0.40).

**Figure 2:**
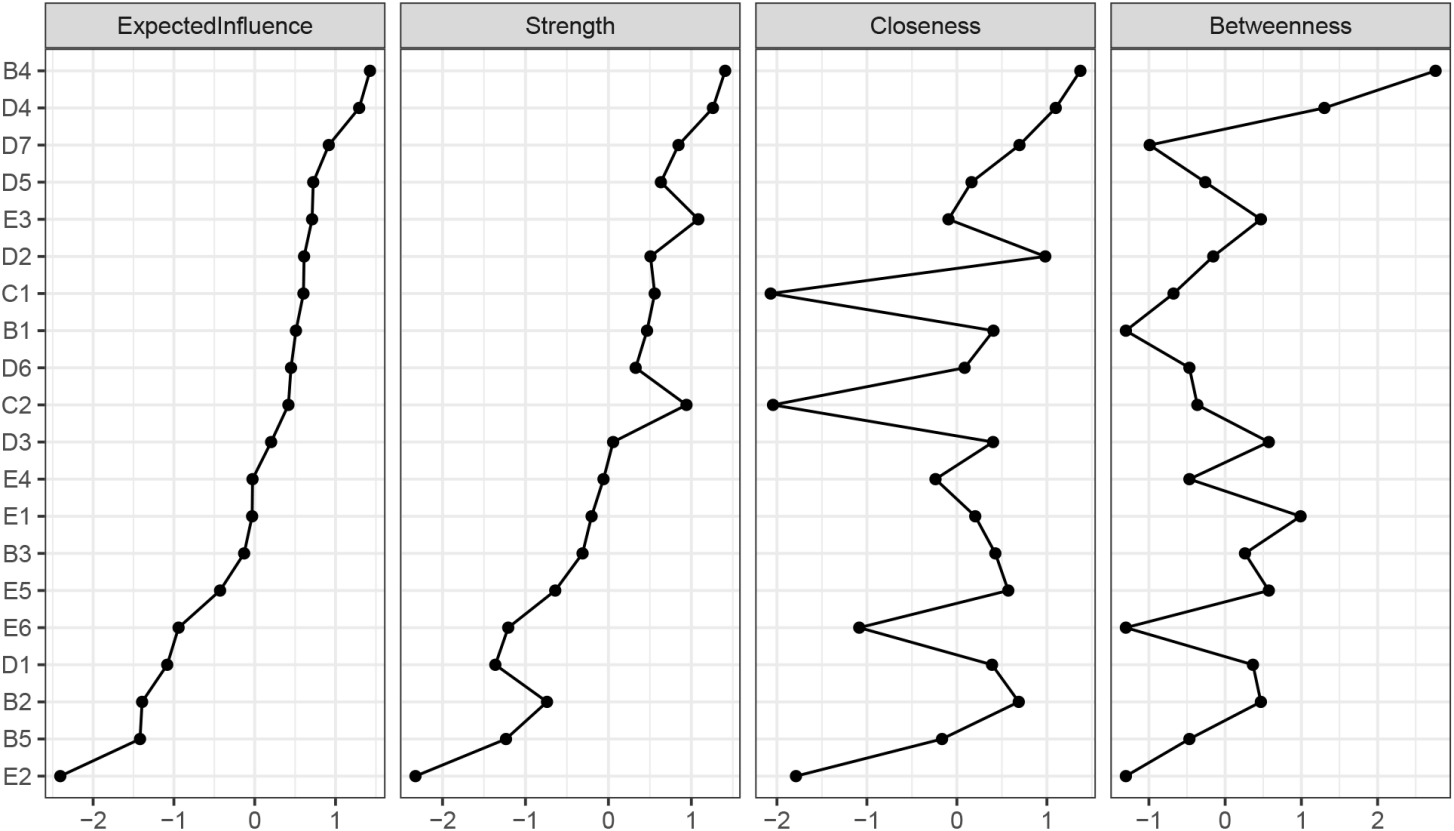
Showing centrality scores for all variables in the network.

**Figure 2** illustrates that emotional cue reactivity (B4) has the highest expected influence (EI =1.18), followed by negative trauma-related emotions (D4) (EI = 1.14) and restricted affect (D7) (EI = 1.05). These results indicate that these symptoms are critical components in the network, while the symptom with the lowest expected influence is self-destructive/reckless behavior (E2) (0.25).

**Figure 3:**
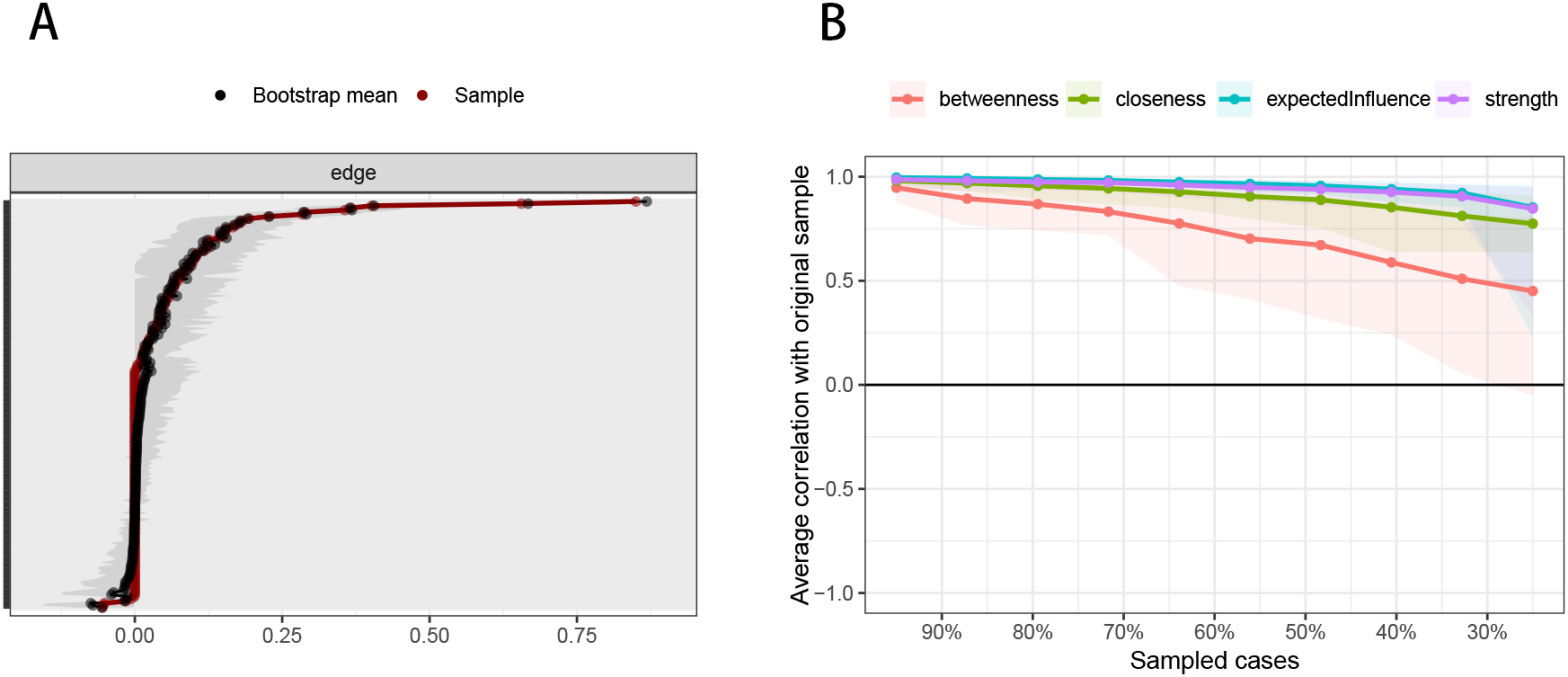
Accuracy and stability of network structure model. **(A)** Accuracy analysis of edge weights. **(B)** Stability analysis of centrality indicators.

**Figure 3A** demonstrates partial overlap between the 95% confidence intervals of the edge weights obtained through non-parametric bootstrapping. At the same time, the absence of overlap in the confidence intervals for some of the strongest edges indicates a relatively precise evaluation of the edge weights. **Figure 3B** shows that the network stability is good, with the stability of expected influence, strength, closeness, and mediation centrality being 0.672, 0.672, 0.594, and 0.283, respectively. A stability coefficient for EI greater than 0.50 indicates that the central symptoms retain stability, even with fewer samples or nodes during network re-estimation.

## 4 Discussion

To our knowledge, this is the first study to conduct a PTSD network analysis among colorectal cancer patients treated with chemotherapy in China. The findings reveal a comparatively compact PTSD network, where symptoms are highly correlated. In our study, strong positive connections often occur among nodes within the same dimension (27–29), which aligns with the concept of PTSD in the DSM-5. The most central symptom is emotional cue reactivity, while the slightest central symptom is self-destructive/reckless behavior.

The results of this study’s symptom network analysis revealed the most vital edge between the symptoms of avoidance of thoughts(C1) and avoidance of reminders(C2), which is in agreement with prior studies (17, 29, 30). PTSD symptoms are often triggered by situational and environmental factors (31). To alleviate their distress, patients who have experienced trauma tend to avoid thoughts, feelings, and places that remind them of the traumatic event, such as recalling the details of the illness or hospital scenes (32). Consequently, the association between avoidance of thoughts and reminders is strengthened. Avoidance is a defense strategy against intense stimuli to temporarily ease perceived distress, accomplishing this by diminishing central nervous system activity (33). However, when avoidance is prolonged, it can lead to heightened adverse cognitive and emotional reactions, ultimately worsening mental states and hindering executive functioning (17).

The strong association between the symptoms of hypervigilance (E3) and exaggerated startle response (E4) aligns with findings from previous PTSD network research (16, 28, 31, 34). Individuals who have been through trauma tend to be more sensitive and vulnerable to triggering events or similar stimuli, a pattern that follows a sensitization model of PTSD (31, 35). As the disease advances, the physical symptoms caused by cancer and its treatment become more severe (36), and patients perceive an intensified threat from cancer (37). Therefore, hypervigilance and exaggerated startle responses reinforce each other (29). Research has shown that trauma-induced prolonged hypervigilance not only disrupts the hippocampus’s ability to function in learning and memory (38) but also impairs emotional and cognitive regulation (17), reducing the person’s ability to face trauma and contributing to the development of PTSD (16, 39).

There is also a strong connection between the symptoms of loss of interest (D5) and detachment (D6), slightly differing from previous studies (40, 41), which found only a moderate association between these symptoms. This discrepancy may relate to the types of trauma examined. For instance, Bryant et al. (41) focused on PTSD resulting from mixed trauma types, such as traffic accidents and assaults, while our research centers on the trauma of a cancer diagnosis. A cancer diagnosis may lead patients to trigger a loss of meaning and purpose in their lives (42), resulting in decreased interest in enjoyable activities, reduced social engagement, and alienation from others (43). As a result, the connection between loss of interest and detachment is strengthened. Additionally, some colorectal cancer patients who have a stoma may feel shame due to changes in their bowel movements, leading them to avoid social interactions (44).

Given the connections between the symptoms above, future healthcare professionals should focus on the associations between avoidance of thoughts and avoidance of reminders, hypervigilance and exaggerated startle response, and loss of interest and detachment. Preventive identification of these vital links can serve as a starting point, combined with early psychological interventions and pharmacotherapy, to weaken the interactions between symptoms, disrupt these strong associations, and improve intervention outcomes. Caregivers should also assist patients in understanding their illness and treatment process, explaining that symptoms such as avoidance and hypervigilance are common stress responses. Techniques like relaxation training and cognitive behavioral therapy (CBT) can alleviate the patients’ psychological stress (45). Patients should be encouraged to participate in social activities actively, rediscover new meanings and goals in life, and rebuild self-confidence, thereby effectively controlling the development of PTSD.

In addition to the edges, emotional cue reactivity (B4) was identified as the most central symptom in the PTSD network of colorectal cancer patients in chemotherapy treatment based on our findings. The core symptom is not aligned with Peters et al. (46), which identified hypervigilance as the central symptom. This variation could stem from the different types of trauma involved, as our research concentrates on patients dealing with the threat of illness. In contrast, Peters et al. examined trauma resulting from an earthquake, a natural disaster. The conditioning theories of PTSD suggest that emotional responses to emotional cue reactivity following exposure to traumatic events are common reactions (47, 48). Individuals develop various associations due to fear, matching trauma-related cues with associated stimuli through classical conditioning (49). As a result, these cues can evoke distressing emotional responses, even in the absence of immediate danger (48). Fear of disease progression in colorectal cancer patients can undermine cognitive abilities and intensify negative beliefs (37). This fear makes it challenging for individuals to shift their attention away from cancer-related cues, leading to increased rumination about these cues (50). Therefore, interventions by healthcare providers aimed at addressing emotional cue reactivity may lead to a more significant reduction in overall PTSD levels. Additionally, future research could explore the influencing factors of this symptom to enable precise interventions, promote psychological recovery, and further reduce PTSD levels.

Interestingly, this study found that the symptom with the lowest centrality is self-destructive/reckless behavior (E2). This finding contrasts with those of most other studies (27, 31, 51), which identified trauma-related amnesia as consistently exhibiting the lowest centrality. This distinction may be attributed to variations in sample size and the interactions between symptoms. Birkeland and Heir noted that intrusive symptoms can lead patients to repeatedly recall their trauma, potentially reducing trauma-related amnesia (52). In our study, intrusive symptoms emerged as the most central symptom, which may have influenced the severity of trauma-related amnesia. Conversely, in the research conducted by Spiller et al. (53), self-destructive/reckless behavior was identified as the most significant symptom. This contradiction may be related to the smaller sample size in Spiller et al.’s study, which may affect the reliability of their findings.

## 5 Strengths and limitations

The advantage of this study is that it is the first to network the PTSD symptoms of colorectal cancer patients undergoing chemotherapy in China. Early identification of these central symptoms may effectively block the progression of other symptoms and reduce the severity of PTSD in patients. Furthermore, given that the PCL-5 is derived from the model of DSM-5, we also observed that the strongly associated clusters (such as C1-C2, E3-E4, D5-D6) are consistent with the conceptual framework of DSM-5, which can provide a supplementary perspective for the classification and multi-dimensional models of mental disorders. Mainly through network analysis methods, it uncovers potential causal relationships between symptoms, providing a valuable reference for promoting patients’ psychological recovery.

Of course, this study also has certain limitations. First, it is a cross-sectional study that provides a fixed view of the PTSD symptom network among chemotherapy-treated colorectal cancer patients, thus failing to capture the dynamic changes of the network over time. Second, we did not carry out subgroup analyses (such as the PTSD group and the non-PTSD group, different age groups, different gender groups, etc.). Therefore, it is difficult for us to determine whether the relationships and patterns observed in the overall sample would still hold in this potentially specific subgroup. Third, the study relied on self-reported questionnaires to assess patients’ PTSD symptoms, which may introduce recall bias. Fourth, the research was conducted solely among colorectal cancer patients in China, so the generalizability of the findings may be limited and should be applied cautiously to other cultural contexts and cancer types. We recommend that future research employ longitudinal data to track the PTSD symptom network among colorectal cancer patients undergoing chemotherapy. Moreover, subgroup analyses ought to be taken into account to offer a more refined perspective. Also, using structured clinical interviews rather than self-reported questionnaires may assist in minimizing recall bias. Subsequently, investigations should be carried out in a wide range of cultural backgrounds and cancer varieties to guarantee the wider generalizability of the results.

## 6 Conclusions

This study reveals the PTSD symptom network structure among colorectal cancer patients undergoing chemotherapy in China. The findings show the most substantial connection between avoidance of thoughts and avoidance of reminders. Moreover, emotional cue reactivity is the most central symptom. We suggest that clinicians should prioritize these central symptoms and their strong interconnections. Targeted interventions based on these network characteristics could improve PTSD management and promote the mental health of patients.

## Data Availability

The original contributions presented in the study are included in the article/supplementary material. Further inquiries can be directed to the corresponding author.
